# Pterostilbene, An Active Constituent of Blueberries, Suppresses Proliferation Potential of Human Cholangiocarcinoma *via* Enhancing the Autophagic Flux

**DOI:** 10.3389/fphar.2019.01238

**Published:** 2019-10-22

**Authors:** Dong Wang, Haoran Guo, Huahong Yang, Dongyin Wang, Pujun Gao, Wei Wei

**Affiliations:** ^1^Department of Hepatology, The First Hospital of Jilin University, Jilin University, Changchun, China; ^2^Institute of Virology and AIDS Research, The First Hospital of Jilin University, Changchun, China; ^3^Key Laboratory of Organ Regeneration and Transplantation of Ministry of Education, Institute of Translational Medicine, The First Hospital of Jilin University, Changchun, China; ^4^Department of Respiration, The First Hospital of Jilin University, Jilin University, Changchun, China

**Keywords:** pterostilbene, human cholangiocarcinoma, autophagy, anti-cancer, light chain 3

## Abstract

**Background:** Human cholangiocarcinoma (CCA) is a highly lethal cancer that occurs in the biliary tract. It is characterized by early invasion, poor outcomes, and resistance to current chemotherapies. To date, an effective therapeutic strategy for this devastating and deadly disease is lacking. Pterostilbene, a natural compound found in the extracts of many plants including blueberries, kino tree, or dragon blood tree, has several health benefits. However, its effects on CCA have not been clarified. Here, we investigated the potential application of pterostilbene for the treatment of human CCA *in vitro* and *in vivo*.

**Methods:** The effects of pterostilbene on CCA cells were determined by assessing cell viability (CCK), cell proliferation, and colony formation. Cell cycle arrest and apoptosis were measured by flow cytometric analysis, whereas proteins related to autophagy were detected by immunofluorescence and immunoblotting assays. A well-established xenograft mouse model was used to evaluate the effects of pterostilbene on tumor growth *in vivo*.

**Results:** Pterostilbene induced dose-dependent and time-dependent cytotoxic effects, inhibited proliferation and colony formation, and caused S phase cell cycle arrest in CCA cells. Instead of triggering apoptotic cell death in these cells, pterostilbene was found to exert potent autophagy-inducing effects, and this correlated with p62 downregulation, elevated expression of endogenous Beclin-1, ATG5, and LC3-II, and increases in LC3 puncta. Pretreating cancer cells with the autophagy inhibitor 3-MA suppressed the induction of autophagy and antitumor activity caused by pterostilbene. Finally, we confirmed that pterostilbene inhibited tumor growth in a CCA xenograft mouse model with minimal general toxicity.

**Conclusion:** Taken together, our findings indicate that pterostilbene, through the induction of autophagic flux, acts as an anti-cancer agent against CCA cells.

## Introduction

Human cholangiocarcinoma (CCA), comprising the malignant transformation of epithelial cells in bile ducts, is the second most common primary hepatic cancer worldwide (Rizvi and Gores, 2013). CCA can occur at multiple positions along the biliary tree. There are many risk factors that are generally recognized as associated with the development of this tumor, such as hepatitis viral infection, parasite infection, biliary stone disease, congenital biliary cysts, primary sclerosing cholangitis, and liver diseases (Labib et al., 2019). In recent years, new cases and the mortality rate of CCA have significantly increased, especially in America and Asia (Saha et al., 2016; Yanala et al., 2019), where the 5-year survival rate is only 15%. Most patients are diagnosed at the advanced stage of disease as it is highly aggressive and not accompanied by specific symptoms. However, it does show early lymph node and distant metastasis (Rizvi et al., 2018). Although surgical resection is the main effective method to treat early-stage CCA, a high recurrence rate is still observed (Squadroni et al., 2017). Furthermore, CCA shows primary resistance to cisplatin and other chemotherapy drugs, which reduces its cure rate (Yu et al., 2018). Therefore, there is an urgent need for novel therapeutic strategies and/or potential effective anticancer agents that can improve the clinical outcomes of CCA.

Pterostilbene, a natural abundant compound in blueberries, kino tree, and dragon blood tree, is a natural methoxylated analogue of the red wine antioxidant, resveratrol (Nagao et al., 2017). Much existing evidence indicates that pterostilbene has therapeutic advantages for cancer prevention and therapy, improving insulin sensitivity, controlling blood glycemia and lipid levels, suppressing cardiovascular diseases, inflammation diseases, and aging, and augmenting memory and cognition (Chen et al., 2018). Recently, pterostilbene has gained increased attention in anti-cancer research as it exhibits hallmark characteristics of an effective anti-cancer agent for different cancers such as human bladder, breast, and colon cancers, leukemia, melanoma, and prostate and stomach carcinomas. The multiple anti-neoplastic mechanisms of pterostilbene exhibit significant overlap among intrinsic and extrinsic apoptotic pathways, cell cycle arrest, DNA damage (Rossi et al., 2013), mitochondrial depolarization, and autophagy. However, the effect of pterostilbene on CCA cell proliferation remains uncertain.

Autophagy is an intracellular catabolic process wherein the lysosome degrades cellular components to provide energy and macromolecular precursors for cell survival (Levy et al., 2017). Defects in autophagy have been related to an increased susceptibility to genomic damage, metabolic stress, and importantly, tumorigenesis (Shacka et al., 2006; Singh et al., 2018). Many anti-cancer agents activate autophagy in different types of cancer cells, thereby conferring tumor cell resistance to chemotherapy. However, excessive autophagy induction in many cancers upon treatment with certain cytotoxic drug or autophagy inducers might lead to autophagic cell death. For instance, combination treatments comprising the anti-cancer drugs temozolomide and dasatinib can suppress glioblastoma cells that are resistant to apoptosis due to autophagy induction (Milano et al., 2009).

In the present study, we investigated the effects of pterostilbene on CCA cells both *in vitro* and *in vivo*, as well as the underlying mechanisms. Pterostilbene dramatically inhibited the viability, migration, and proliferation of CCA cells and induced CCA cell cycle arrest at the S phase. Importantly, the cytotoxicity of pterostilbene towards CCA cells was dependent on autophagy induction, which triggered autophagic cell death, rather than apoptosis. We confirmed that pterostilbene suppresses tumor growth in a CCA xenograft mouse model without serious toxic reactions. Therefore, our findings demonstrate that pterostilbene has potential clinical value for CCA therapy.

## Materials and Methods

### Cell Lines and Compound Preparation

RBE and HCCC-9810 cells were obtained from the Type Culture Collection of the Chinese Academy of Sciences (Shanghai, China) and grown in RPMI-1640 medium (Biological Industries, IL) supplemented with 10% fetal bovine serum (PAN Biotech, SA) and 1% penicillin/streptomycin solution (TransGen Biotech, Beijing, China) at 37 °C in an incubator containing 5% CO_2_. Pterostilbene (N2126, purity = 99.94%) was purchased from APExBIO (USA) and stored at −20 °C. Z-VAD-FMK (HY-16658, purity = 98.20%) was purchased from MedChemExpress (Monmouth Junction, NJ, USA). 3-methyladenine (3-MA, M9281, purity >98.00%) was purchased from Sigma-Aldrich (St. Louis, MO, USA).

### Cell Proliferation Assay

Cell viability was determined using a Cell Counting Kit (CCK; TransGen Biotech, FC101-02). Cells were seeded in 96-well plates at a density of 3,000 cells/well and then incubated in medium containing 10 µl CCK for 2 h at 37°C after treating them with serial dilutions of pterostilbene for 24, 48, and 72 h. Absorbance was detected using a microplate reader (Bio-Rad Laboratories, Inc. Hercules, CA, USA) at a wavelength of 450 nm.

### Cell Viability Assay

For cell growth assays, 2 × 10^4^ cells per well were seeded in 24-well plates, cultured for 24 h, and treated with DMSO or pterostilbene at 30, 60, and 120 µM for 24, 48, and 72 h. After trypsinization and staining with trypan blue, cells were counted with an optical microscope using a hemocytometer at the indicated time-points. All experiments were repeated three times.

### Cell Clonogenic Assay

HCCC-9810 and RBE cells were plated in 6-well plates at a density of 800 cells/well. Cells were grown for 24 h and then treated with pterostilbene for 14 days. Cells were then washed with phosphate-buffered saline (PBS), fixed with 100% methanol for 30 min, and stained with 0.02 g/ml crystal violet solution in 20% ethanol. Colonies were examined by phase-contrast microscopy (Olympus), and colonies with more than 50 cells were counted. All experiments were repeated three times.

### Transmission Electron Microscopy

Cells were fixed in 2.5% glutaraldehyde (pH 7.4) at 4°C overnight. After fixation, cells were rinsed in 0.1 M phosphate buffer and centrifuged. After fixation with 1% osmium tetroxide, the pellets were dehydrated in a series of preparations of graded acetone and embedded in epoxy resin. Electron micrographs of randomly-selected thin sections were taken using a JEM-1400 plus transmission electron microscope (JEOL, Japan).

### Cell Cycle Assay

HCCC-9810 and RBE cells were collected with trypsin solution after pterostilbene treatment for 48 and 72 h. The cells were washed twice with PBS, fixed in pre-cooled 75% ethanol at 4°C overnight, and then incubated with propidium iodide (PI; Yuanye, Shanghai, China, R20288) for 1 h at room temperature in the dark. A fluorescence-activated cell sorting (FACS) flow cytometer (BD Bioscience) was used for cell cycle analysis.

### Apoptosis Assay

Apoptotic cells were identified and quantified using an Annexin V-FITC Apoptosis Kit (BD Biosciences, 556547). After pterostilbene treatment for 48 h, cells were digested with trypsin solution without EDTA and washed with ice-cold PBS twice. Cells were then resuspended in 1× binding buffer to 1 × 10^5^ cells/100 μl medium. Cells were incubated with Annexin V-FITC and PI for 15 min at room temperature in the dark. After the addition of 400 μl of 1× binding buffer to each tube, samples were evaluated by fluorescence-activated cell sorting using a flow cytometer (BD Bioscience). Data were analyzed with CellQuest Pro Software (BD Bioscience).

### Immunoblotting Assay

Cell lysates were prepared in lysis buffer [150 mM tris (pH 7.5) with 150 mM NaCl, 1% triton X-100, and complete protease inhibitor cocktail tablets (Roche)] and 1× loading buffer (TransGen Biotech). Samples were separated by sodium dodecyl sulfate polyacrylamide-gel electrophoresis and transferred to nitrocellulose membranes using a semi-dry apparatus (Bio-Rad). After blocking with 5% nonfat milk, the membranes were probed overnight with appropriately diluted primary antibodies. The membranes were then washed and incubated with alkaline phosphatase-conjugated secondary antibodies at room temperature for 1 h. Various primary antibodies against the proteins of interest were used, including those targeting Cyclin A2, Cyclin E1, ATG5, Beclin1, light chain 3 (LC3), and p62, which were purchased from Proteintech Group, Inc. (Chicago, IL, USA). Anti-β-actin mouse monoclonal antibodies was purchased from GenScript Biotech Corp. (NJ, USA). The secondary antibodies were goat anti-rabbit IgG (H+L) and goat anti-mouse IgG (H+L) (Jackson ImmunoResearch Laboratories). Staining was conducted using a BCIP/NBT Alkaline Phosphatase Color Development Kit (Zoman Biotechnology Co., Ltd., Beijing, China).

### Immunofluorescence Assays

Cells were seeded on glassy plates (cat. no. 801001; Nest) and treated with pterostilbene and/or 3-MA for 48 h. After fixation in 4% paraformaldehyde for 30 min, cells were permeabilized with 0.2% triton X-100 for 10 min, blocked with goat serum (cat. no. ZLI-9022; ZSGB-BIO) for 1 h, and incubated overnight with anti-LC3 antibodies at a dilution of 1:100. Alexa Fluor 488-conjugated goat anti-rabbit IgG (cat. no. A-11034; Life Technologies) was used as the secondary antibody. The nuclei were counterstained with DAPI (10 μg/ml; cat. no. top0221; Biotopped). Images were captured using a confocal microscope (LSM 880; Carl Zeiss, Germany).

### *In Vivo* Xenograft Experiment

A total of 1 × 10^7^ HCCC-9810 cells were suspended in 100 µl of serum-free medium and mixed with an equal volume of Matrigel (Corning, USA), and then subcutaneously injected into the dorsal flanks of 4-week-old BALB/C–NU mice (Beijing Vital River Laboratory Animal Technology Co, Ltd). After tumors reached approximately 200 mm^3^, mice were randomly assigned to the pterostilbene-treated group (30 and 60 mg/kg) or control group. The pterostilbene-treated group received intraperitoneal injections of pterostilbene (30 and 60 mg/kg) or vehicle (2.5%DMSO in 100 μl PBS) once every 2 days for 3 weeks, while the control group received vehicle control of equal volume. Tumor volume was measured with calipers every 2 days and calculated using the following equation: V = L × W^2^/2, where L represents tumor length and W represents tumor width. Finally, the tumors and organs from mice in the three groups were collected and used to perform histological analysis based on H&E staining. This study was reviewed and approved by the *Animal Welfare and Research Ethics Committee at Jilin University*.

### Statistical Analysis

All data were analyzed using GraphPad Prism 6 (GraphPad Software, Inc., San Diego, CA, USA) and presented as the mean ± standard deviation. Differences among test groups were analyzed by ANOVA. P values less than 0.05 were considered statistically significant.

## Results

### Pterostilbene Inhibits the Proliferation and Clonogenicity of Human CCA Cells

**Figure 1:**
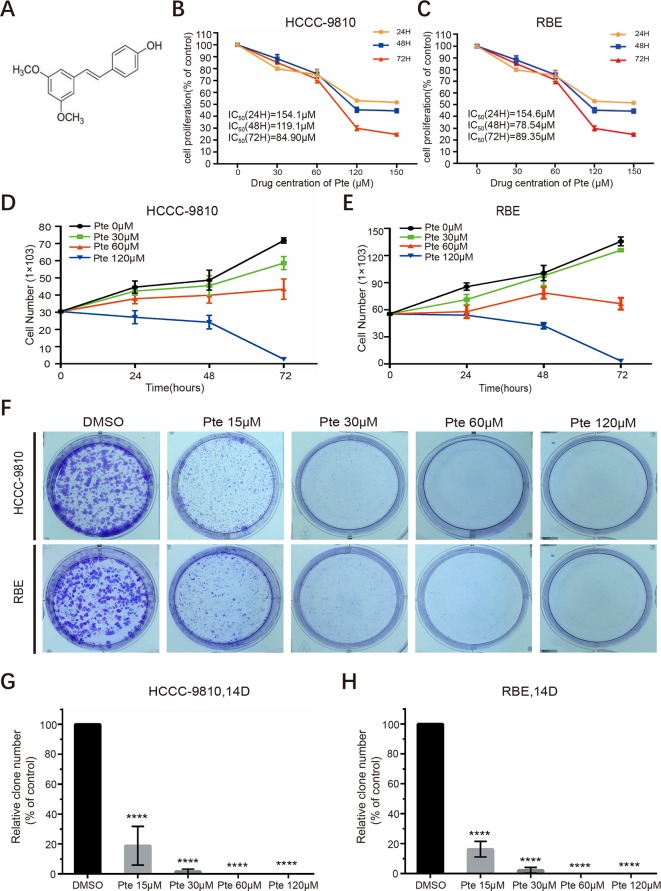
Pterostilbene inhibits the growth of cholangiocarcinoma cancer cells. **(A)** Chemical structure of pterostilbene (Pte). **(B**, **C)** Pterostilbene reduces cholangiocarcinoma proliferation. The proliferation of cholangiocarcinoma cells was determined by CCK assays after treatment with serial dilutions of pterostilbene for 24, 48, and 72 h (n = 3). H, hour. **(D**, **E)** Pterostilbene inhibited cholangiocarcinoma viability. Cells were seeded in a 24-well plate, incubated at 37°C in a 5% CO_2_ incubator, treated with DMSO or pterostilbene (30, 60, and 120 µM), trypsinized for different periods of time, and stained with trypan blue and counted (n = 3). **(F**–**H)** Pterostilbene suppressed cholangiocarcinoma cancer cell colony formation. Eight hundred cells were seeded into a 6-well plate in 2 ml of medium, treated with different concentrations of pterostilbene, and incubated at 37°C in a 5% CO_2_ incubator for 14 days, followed by Giemsa staining and cell colony (> 50 cells) counting (****P < 0.0001, n = 3). D, day.

Pterostilbene (trans-3,5-dimethoxy-4-hydroxystilbene) is an analog of the well-studied resveratrol but is significantly more bioavailable (**Figure 1A**). We first assessed the anti-proliferative effects of pterostilbene in CCA cells. HCCC-9810 and RBE cells were treated with increasing concentrations of pterostilbene (0, 30, 60, 120, 150 µM) for 24, 48, and 72 h. Unlike that in the control group, pterostilbene significantly inhibited CCA cell proliferation in a dose-dependent manner. Moreover, this inhibition was more evident as time progressed (**Figures 1B, C**). We then explored the effect of pterostilbene at concentrations of 0, 30, 60, 120 µM on the suppression of CCA cell viability *in vitro* and confirmed that treatment with pterostilbene time- and dose-dependently decreased the numbers of HCCC-9810 and RBE cells (**Figures 1D, E**). This result indicated that pterostilbene has strong cytotoxic effects on the CCA cell lines.

We proceeded to perform clonogenic assays to determine the long-term anti-proliferative effects of pterostilbene towards HCCC-9810 and RBE cells. Our results showed that pterostilbene treatment strongly inhibited clone formation for both CCA cells in a dose-dependent manner (0,15, 30, 60, 120 µM) (**Figures 1F–H**). We also noted that pterostilbene remarkably decreased the clone numbers of both CCA cell lines at a low concentration (15 µM), which might have been due to the low cell density used in this assay, which increased sensitivity to the anti-CCA activity of pterostilbene. Together, these findings reveal that pterostilbene efficiently reduces the growth of CCA cells.

### Pterostilbene Induces Cell Cycle Arrest in the S Phase in CCA Cells

**Figure 2:**
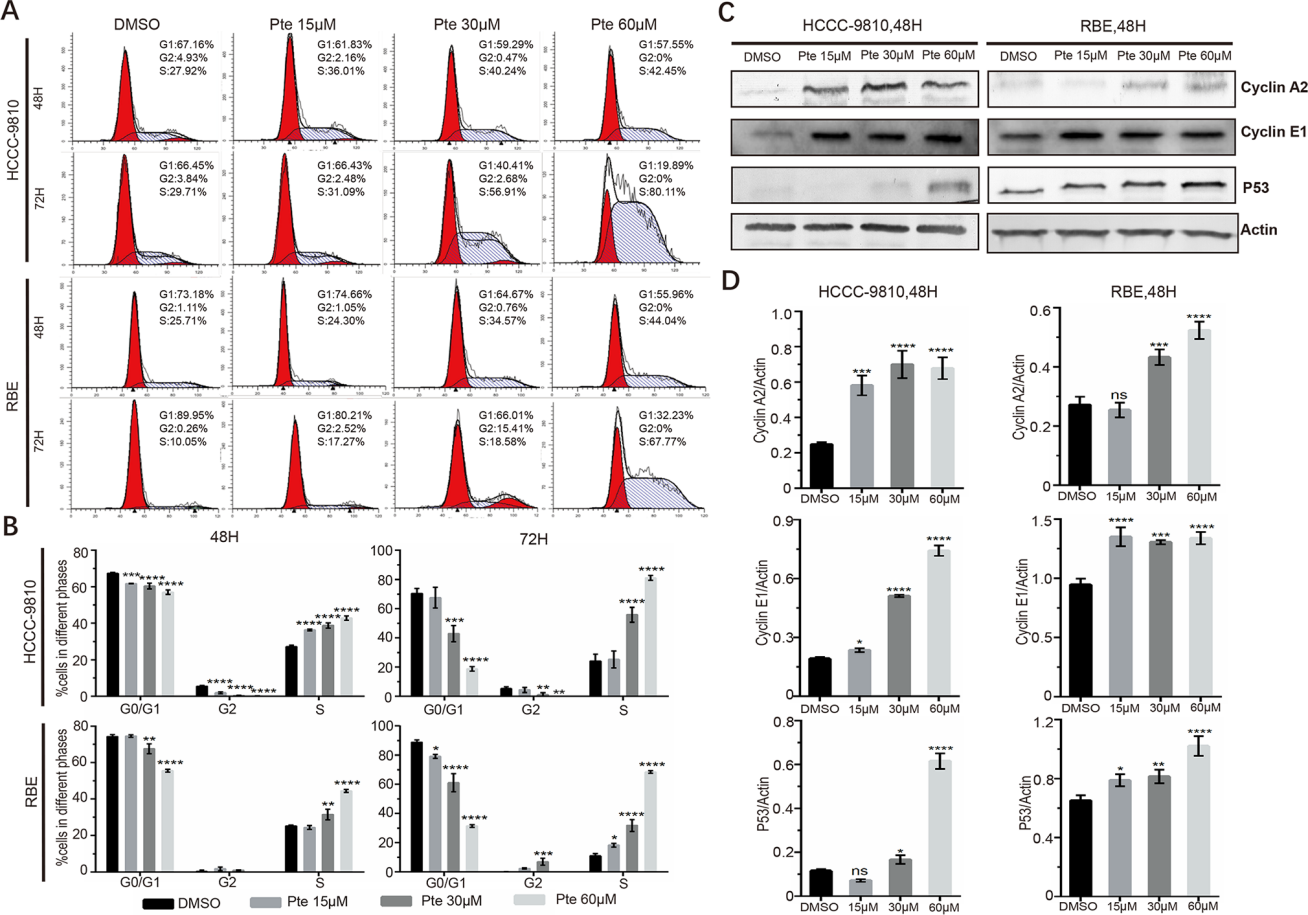
Pterostilbene induces S cell-cycle arrest in cholangiocarcinoma cancer cells. **(A**, **B)** Cells were collected with trypsin solution after pterostilbene treatment for 48 and 72 h, incubated with propidium iodide, and analyzed by flow cytometry. **(C**, **D)** Cyclin A2, Cyclin E1, and P53 were detected by immunoblot analysis. Cells were treated with DMSO or pterostilbene for 48 h (*P < 0.05, **P < 0.01, ***P < 0.001, ****P < 0.0001, n = 3).

To further elucidate whether the effects of pterostilbene on the growth of CCA cells are mediated by the inhibition of cell cycle progression, HCCC-9810 and RBE cells were treated with 15, 30, and 60 µM pterostilbene for 48 and 72 h. By propidium iodide staining-dependent flow cytometric assays, we found that pterostilbene treatment markedly increased the accumulation of both cell lines at the S phase compared to that observed in vehicle-treated cells (**Figures 2A, B**). Consistent with this result, pterostilbene treatment resulted in an evident increase in the expression of cyclin proteins at S phase including cyclin A2 and cyclin E1 in both HCCC-9810 and RBE cells (**Figures 2C, D**). Moreover, we found that expression levels of the tumor suppressor p53 in CCA cells were elevated in the presence of pterostilbene (**Figures 2C, D**). Hence, cell cycle arrest might serve as one of the mechanisms of the anti-tumor activity of pterostilbene.

### Pterostilbene Induces Cytoplasmic Vacuolation in CCA Cell Lines in a Caspase- and Apoptosis-Independent Manner

**Figure 3:**
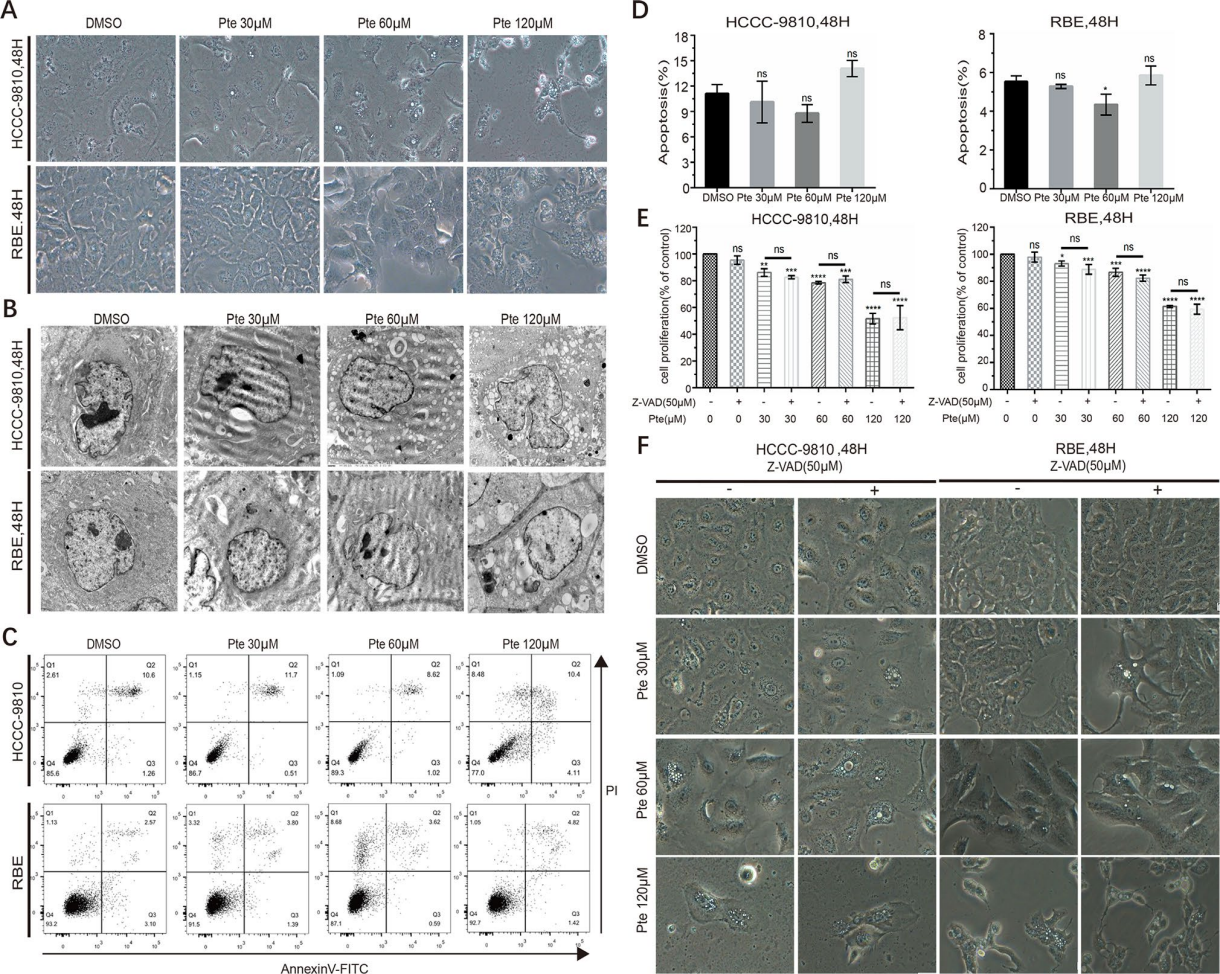
Pterostilbene induces cytoplasmic vacuolation in cholangiocarcinoma (CCA) cell lines in a caspase- and apoptosis-independent manner. **(A)** HCCC-9810 and RBE cells were treated with 30, 60 and 120 µM pterostilbene for 48 h. Microscopic observation revealed the formation of vacuoles in the cytoplasm of treated cells. **(B)** Transmission electron micrograph (10,000×) of HCCC-9810 and RBE cells treated with 30, 60, and 120 µM pterostilbene for 48 h. Inset shows cell with larger vacuoles after 48 h treatment with pterostilbene. **(C**, **D)** HCCC-9810 and RBE cells were treated with different concentrations of pterostilbene for 48 h, stained with Annexin V-FITC and PI, and then measured by flow cytometry. The percentages of cells in early- and late-state apoptosis, as well as total apoptotic cells, after treatment with 0, 30, 60 and 120 µM of pterostilbene, compared to those in control cells, are shown as mean ± SD of three independent experiments. **(E)** Histograms show the viability of CCA cells treated with DMSO, pterostilbene (30, 60, 120 µM), and/or Z-AVD-FMK for 48 h. Cell viability was determined by a CCK assay after 48 h. **(F)** Images (200×) show that Z-VAD could not change pterostilbene-induced cytoplasmic vacuolation in cells. Data represent the mean ± SD (**P < 0.01, ***P < 0.001, ****P < 0.0001, n = 3).

In pterostilbene-treated CCA cells, we observed dramatic morphological changes at concentrations of 30, 60 µM and 120 µM (**Figure 3A**). However, in contrast to previous studies where pterostilbene induced apoptosis in distinct cancer cells, we did not detect any highly characteristic features of apoptosis such as cytoplasmic shrinkage and nuclear fragmentation, in pterostilbene-treated CCA cells. Interestingly, we observed an increase in vacuole formation in the cytoplasm of CCA cells in the presence of pterostilbene by either light or electron microscopy (**Figures 3A, B**). By annexin V-FITC/PI-labeled flow cytometric assay to further eliminate the effects of pterostilbene on the induction of CCA cell apoptosis, such treatment was found to have no significant influence on apoptotic rates of CCA cells (**Figures 3C, D**). To support our conclusion, we confirmed that an apoptosis suppressor, Z-VAD, a pan-caspase inhibitor, did not reverse the anti-tumor activity of pterostilbene in both CCA cell lines (**Figure 3E**). Moreover, Z-VAD failed to interrupt pterostilbene-induced morphological changes and vacuole accumulation (**Figure 3F**). Thus, the suppression of CCA cell growth by pterostilbene does not rely on apoptosis induction.

### Autophagy Is Involved in the Anti-Cancer Effects of Pterostilbene Toward CCA Cells

**Figure 4:**
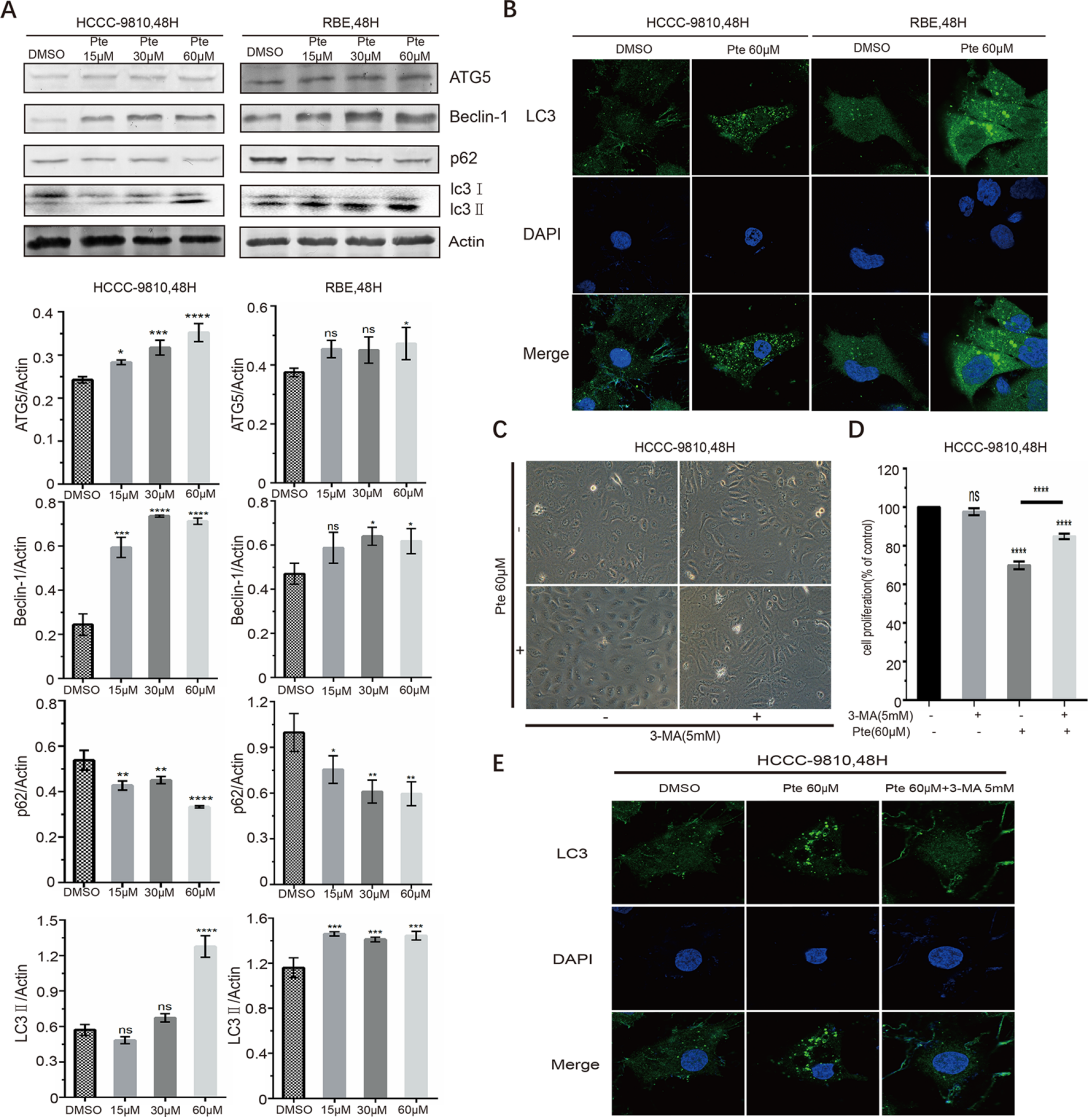
Effects of pterostilbene on autophagy-associated signals in cholangiocarcinoma cancer cells. **(A)** Cells were treated with 0, 15, 30, and 60 µM pterostilbene for 48 h, and then protein lysates were individually prepared. ATG5, Beclin-1, p62, and LC3, were detected by western blotting. Representative images were taken from three independent experiments. **(B)** After treatment with pterostilbene for 48 h, HCCC-9810 and RBE cells were stained for LC3 and analyzed for intracellular distribution by confocal microscopy. An overlay of blue (nuclei) and green (LC3) channels is shown. **(C)** Images (200×) show that 3-MA could suppress the pterostilbene-induced cytoplasmic vacuolation. **(D)** Cell proliferation showing that the autophagy inhibitor 3-MA could reverse the toxicity of pterostilbene towards HCCC-9810 cells. **(E)** Co-treatment with pterostilbene and 3-MA inhibited endogenous LC3 puncta formation in HCCC-9810 cells (*P < 0.05, **P < 0.01, ***P < 0.001, ****P < 0.0001, n = 3).

Pterostilbene has been found to cause autophagy in other tumor cells (Wang et al., 2012; Ko et al., 2015; Kang et al., 2019; Yu et al., 2019); however, there is no evidence of whether pterostilbene induces autophagy in CCA cells. Here, we measured the expression of autophagy marker proteins in HCCC-9810 and RBE cells. Immunoblotting data indicated that CCA cells treated with increasing concentrations of pterostilbene had elevated expression levels of ATG5 and Beclin-1 proteins, which are essential for the autophagy pathway (**Figure 4A**). Moreover, LC3 is a hallmark of autophagy and the conversion of LC3-I to LC3-II *via* proteolytic cleavage and lipidation indicates autophagy induction. We detected a marked increase in the ratio of LC3-II/LC3-I in pterostilbene-treated cells compared to that in the control group (**Figure 4A**). Meanwhile, p62 protein, which can be efficiently degraded by autophagy, was downregulated in both CCA cell lines in the presence of pterostilbene (**Figure 4A**). LC3-II is essential for the formation of autophagosomes and the completion of macroautophagy. We sought to validate the effects of pterostilbene on the formation of LC3 puncta in CCA cells by performing immunofluorescence assays. We also observed the induction of endogenous LC3 puncta in CCAs exposed to pterostilbene at a concentration of 60 µM (**Figure 4B**). These results indicated that pterostilbene efficiently triggers autophagy in CCA cells.

Importantly, when CCA cells were treated with a combination of pterostilbene and an autophagy inhibitor, namely 3-MA, pterostilbene-induced cytoplasmic vacuolation was markedly suppressed and the cells displayed mild morphological changes compared to those observed in pterostilbene single-treated CCAs (**Figure 4C**). Moreover, we found that 3-MA counteracted the cytotoxic effects of pterostilbene on CCAs (**Figure 4D**). Co-treating CCA cells with pterostilbene and 3-MA inhibited endogenous LC3 puncta formation (**Figure 4E**), indicating that 3-MA blocked pterostilbene-activated autophagy. Autophagy induction by pterostilbene is therefore critical for its anti-cancer activity as it suppresses cell viability and enhances cytoplasmic vacuolation in CCA cells.

### Pterostilbene Suppresses the Growth of HCCC-9810 Xenografts *In Vivo*

**Figure 5:**
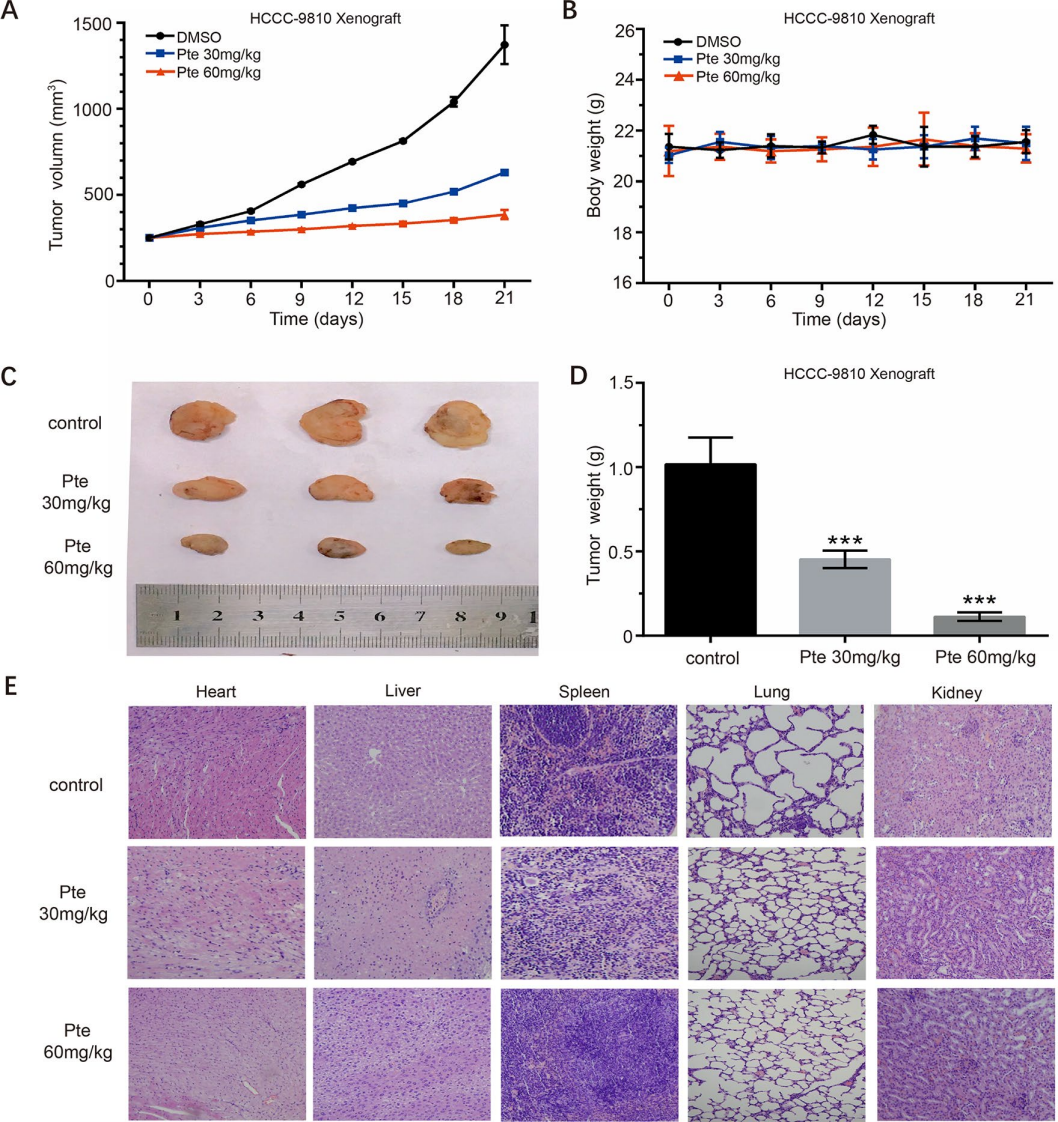
Pterostilbene suppresses the *in vivo* growth of the HCCC-9810 xenograft model established with HCCC-9810 cells. When xenograft tumors reached a size of approximately 200 mm^3^, mice were randomized into three groups. **(A)** Tumor volumes and **(B)** body weights of tumor-bearing nude mice in vehicle (DMSO), pterostilbene (30 mg/kg), and pterostilbene (60 mg/kg) groups. **(C)** Final volumes and **(D)** weights of tumors after treatment (***P < 0.001). **(E)** H&E staining of organs collected from tumor-bearing nude mice after treatment.

We used CCA xenografts in BALB/C–NU mice to further investigate the anti-tumor activity of pterostilbene *in vivo*. HCCC-9810 cells were subcutaneously injected into the backs of mice. When palpable xenograft tumors grew to 200 mm^3^, mice were randomly assigned to either the control or treatment group. Over time, xenograft tumors in the treatment group progressed slower than those in the control group (**Figure 5A**). Our results showed that tumor growth in the two treatment groups was significantly inhibited in a dose-response manner compared to that in the control group. In addition, mice did not show any obvious signs of toxicity after the entire treatment (**Figures 5B, E**) as described in a previous study (Mei et al., 2018). On the 21st day, all tumors were collected. The sizes of xenograft tumors in the treatment group were much smaller than those of the control group (**Figure 5C**). Further, the weights of xenograft tumors aligned with their consequent sizes (**Figure 5D**). These results suggest that pterostilbene significantly inhibits the growth of HCCC-9810 xenografts *in vivo*.

## Discussion

CCA is considered a primary liver cancer. Although its incidence is lower than that of hepatocellular carcinoma (HCC), its prognosis is much worse. CCA is a devastating cancer showing resistance to most known chemotherapeutic drugs; systemic chemotherapy is however the standard treatment (Banales et al., 2016; Chao-En et al., 2019). Hence, developing natural compounds as novel chemo-preventative agents has become a very promising option in the study of CCA treatment and therapy. Here, we showed the significant anti-cancer effect of pterostilbene on CCA cells both *in vitro* and *in vivo via* the induction of cell-cycle arrest and autophagic cell death.

Pterostilbene, a natural dimethylated analog of resveratrol, has been shown to exert a wide range of biological effects on human health, including anti-oxidative, anti-inflammatory, and anti-cancer (Rimando et al., 2002). An important advantage of pterostilbene, relative to resveratrol, is its higher bioavailability. Moreover, its better lipophilic and oral absorption, higher cellular uptake, and longer half-life than resveratrol, make it a good prospect for future clinical applications. However, there was previously no data on the effects of pterostilbene on human CCA and its underlying mechanisms. The identification of pterostilbene as a novel anti-CCA agent should be helpful to accelerate its entry into clinical trials for CCA treatment.

In this preclinical study, we found that pterostilbene is a potent inhibitor of CCA cell proliferation *in vitro* and *in vivo*. Treatment with pterostilbene suppressed the growth and viability of CCA cells in a time- and dose- dependent manner. The clonogenicity of these cells was also repressed by pterostilbene (**Figure 1**), and tumor growth of CCA xenografts in athymic nude mice *in vivo* was also suppressed (**Figure 5**). As aberrant activity of various cell cycle proteins results in uncontrolled cancer cell proliferation, cell cycle regulators are regarded as attractive targets for oncotherapy. Herein, our data demonstrated that pterostilbene treatment could trigger cell cycle arrest at the S phase in CCA cells. In addition, we detected a dose-dependent elevation of the expression levels of cyclin A2 and cyclin E1 in pterostilbene-treated CCAs (**Figure 2**). Previous studies revealed that pterostilbene induces G1 phase arrest at high doses (usually greater than 50 µM) and S phase arrest at low concentrations (Lee et al., 2016); however, we did not detect such phenomena in pterostilbene-treated CCAs. As the mechanisms whereby pterostilbene induces cell cycle arrest at different phases in different cancer cells remain unclear, a further investigation is warranted. Nonetheless, we noticed that pterostilbene treatment significantly increased the expression of p53 in HCCC-9810 and RBE cells, which should contribute to the maintenance of cell cycle checkpoints and the blockage of abnormal mitosis.

Apoptosis, a well-known mechanism of programmed cell death, is considered a natural barrier during tumorigenesis and an important therapeutic target. Pterostilbene has been confirmed to induce apoptosis in numerous cancer cells including lung, prostate, bladder, colon, cervical, melanoma, and breast (Ferrer et al., 2005; Chen et al., 2012; Nikhil et al., 2014; Kala et al., 2015; Dhar et al.,2016; Chatterjee et al., 2018). Pterostilbene can promote cancer cell apoptosis through the intrinsic pathway, extrinsic pathway, or endoplasmic reticulum stress pathway in different cancer cells. However, our study indicated that pterostilbene did not induce apoptosis in CCA cells. Combining pterostilbene with the pan-caspase inhibitor, Z-VAD, had no effect on the cytotoxicity of pterostilbene towards CCA cells (**Figure 3**), thereby suggesting that pterostilbene exerts distinct anti-cancer effects, rather than the induction of apoptosis, on CCA cells.

Autophagy, another type of cell death, is a process wherein unnecessary or dysfunctional cytoplasmic components are degraded to maintain cellular homeostasis for normal physiologic functions (Klionsky et al., 2012). Numerous studies have reported the autophagy-inducing property of pterostilbene with respect to various tumor cells. The microtubule-associated protein LC3 is a key autophagy regulator that is distributed abundantly in the nucleus and cytoplasm. Upon the initiation of autophagy, a key autophagy regulator, LC3B, is cleaved by ATG4 to produce LC3B-I, and subsequently conjugated with phosphatidylethanolamine to generate LC3-II by the ATG5–ATG12/ATG16 complex (Lorente et al., 2018). In the present study, we observed that pterostilbene treatment might significantly elevate the expression of ATG5 and another autophagy protein, Beclin-1. The ratio of LC3-II to LC3-I was also increased. The well-defined molecule p62, which undergoes turnover during autophagy, was decreased in pterostilbene-treated CCA cells. Furthermore, immunofluorescence assays showed that pterostilbene treatment resulted in the dramatic accumulation of LC3 puncta, which is a classic phenomenon associated with autophagy activation. Thus, pterostilbene is a potent inducer of autophagy in CCA cells.

In cancer cells, autophagy has been documented to play a dual role, mediated by both pro-survival and pro-death mechanisms, based on the cancer type, stage, and genetic context. However, we demonstrated that autophagy inhibition *via* a common autophagy inhibitor, namely 3-MA, impaired pterostilbene-induced morphological changes, cytoplasmic vacuolation, cytotoxicity, and LC3-II puncta accumulation in CCA cells (**Figure 4**). Thus, the anticancer activity of pterostilbene towards CCA cells could rely on enhanced autophagic flux. As the mechanisms whereby pterostilbene-induced autophagy contributes to the suppression of cancer cell proliferation but does not facilitate cell survival in CCA cells remain unclear, further investigation is needed. The results further add to the potential of pterostilbene for the treatment of autophagy-dependent resistance to chemotherapy in cancer cells, which should be investigated in future studies.

CCA is a highly-malignant neoplasm with poor prognosis and no effective therapy as a high level of drug resistance usually diminishes the efficacy of chemotherapy drugs. In the present study, we showed that pterostilbene significantly inhibited CCA growth without causing serious toxic effects over the entire experimental period in the mouse CCA HCCC-9810 xenograft tumor model (**Figure 5**). This finding supports the contention that pterostilbene might have broad applications for CCA treatment.

To summarize, pterostilbene induced dose-dependent and time-dependent cytotoxicity, anti-proliferative effects, cell-cycle arrest at the S phase, and caspase-independent autophagic death in CCA cells. These findings shed light on a novel strategy and indicate the value of pterostilbene for the treatment of CCA, which should be addressed in future clinical investigations.

## Data Availability Statement

All datasets generated for this study are included in the manuscript/supplementary files.

## Ethics Statement

The animal study was reviewed and approved by the Animal Welfare and Research Ethics Committee at Jilin University.

## Author Contributions

DW, HG, HY, and DyW performed the experiments. DW, HG, HY, and WW analyzed the data. WW, DW, and PG wrote the paper with help from all authors. WW and PG directed the project.

## Conflict of Interest

The authors declare that the research was conducted in the absence of any commercial or financial relationships that could be construed as a potential conflict of interest
